# Two-dimensional ultrathin gold film composed of steadily linked dense nanoparticle with surface plasmon resonance

**DOI:** 10.1186/1556-276X-7-683

**Published:** 2012-12-21

**Authors:** Long-De Wang, Tong Zhang, Sheng-Qing Zhu, Xiao-Yang Zhang, Qi-Long Wang, Xuefeng Liu, Ruo-Zhou Li

**Affiliations:** 1 School of Electronic Science and Engineering and Key Laboratory of Micro-Inertial Instrument and Advanced Navigation Technology, Ministry of Education, Southeast University, Nanjing, 210096, People’s Republic of China; 2 Suzhou Key Laboratory of Metal Nano-Optoelectronic Technology, Suzhou Research Institute of Southeast University, Suzhou, 215123, People’s Republic of China; 3 Institute of Optics and Electronics, CAS, PO Box 350, Shuangliu, Chengdu, 610209, China; 4 Department of Chemistry and Chemical Engineering, Huainan Normal University, Huainan, 232001, People’s Republic of China

**Keywords:** Ultrathin gold film, Surface plasmon resonance, SERS

## Abstract

**Background:**

Noble metallic nanoparticles have prominent optical local-field enhancement and light trapping properties in the visible light region resulting from surface plasmon resonances.

**Results:**

We investigate the optical spectral properties and the surface-enhanced Raman spectroscopy of two-dimensional distinctive continuous ultrathin gold nanofilms. Experimental results show that the one- or two-layer nanofilm obviously increases absorbance in PEDOT:PSS and P3HT:PCBM layers and the gold nanofilm acquires high Raman-enhancing capability.

**Conclusions:**

The fabricated novel structure of the continuous ultrathin gold nanofilms possesses high surface plasmon resonance properties and boasts a high surface-enhanced Raman scattering (SERS) enhancement factor, which can be a robust and cost-efficient SERS substrate. Interestingly, owing to the distinctive morphology and high light transmittance, the peculiar nanofilm can be used in multilayer photovoltaic devices to trap light without affecting the physical thickness of solar photovoltaic absorber layers and yielding new options for solar cell design.

## Background

Noble metal nanoparticles and nanofilms with strong localized surface plasmon resonances (LSPRs) have attracted great interests in fields such as nanoscale photonics, biological sensing [1,2], surface-enhanced Raman scattering (SERS) [3,4], photocatalytic and photoelectrochemical processes [5], and plasmonic absorption enhancement in solar cells [6-19]. The LSPRs arise from the excitation of a collective electron oscillation within the metallic nanostructure induced by the incident light, leading to enormous optical local-field enhancement and a dramatic wavelength-selective photon scattering at the nanoscale [20-23]. The exceptional optical properties introduced by LSPRs have spurred tremendous efforts to design and fabricate highly SERS-active substrates for molecular sensing. The most studied and best established systems are substrates sprayed with Ag or Au colloids that give high SERS signals at some local ‘hot junctions’ [24]. In order to fabricate noble nanoparticle arrays with high SERS activity and improve the uniformity, lithographic techniques have been employed.

We have recently reported a relatively simple approach in fabricating uniform gold nanocrystal-embedded nanofilms via a conventional magnetron sputtering method. In this method, one can more conveniently assemble noble metals with precise gap control in the sub-10-nm regime [25] than any other method. As a continual effort in supporting the above claim, here we report further evidence such as visible absorption spectra of the Au film on indium tin oxide (ITO) glass substrates, the blend films of poly(3,4-ethylenedioxythiophene) doped with poly(styrenesulfonate) (PEDOT:PSS) and poly(3-hexylthiophene) and [6,6]-phenyl-C_61_-butyric acid methyl ester (P3HT:PCBM) on ITO glass substrates, and the SERS measurements of molecules adsorbed on gold nanocrystals deposited on ITO glass substrates. Our results suggest that the continuous ultrathin nanofilm can obviously enhance visible-range absorption in the active layer of solar cells and obtain an ultrasensitive SERS-active coating.

## Methods

### The fabrication of continuous ultrathin Au nanofilms

Our approach is based on the formation of Au nanofilms on the buffer layer surface of PEDOT:PSS or on ITO glass utilizing magnetron sputtering deposition of metal atoms. The ITO-coated glass substrate was first cleaned with detergent, then ultrasonicated in acetone and isopropyl alcohol for further cleaning, and subsequently dried in a vacuum oven at 80°C for 3 h. PEDOT:PSS films with thicknesses of 30 nm are prepared via spin coating on top of the ITO glass and cured at 130°C for 10 min in air. On top of the freshly prepared PEDOT:PSS layer, metallic gold are sputtered by magnetron sputtering in an electrical current of 0.38 A, vacuum of 0.15 Pa, Ar flux of 25 sccm, and discharge of 1 s. The ITO/Au nanofilm is fabricated in an identical magnetron sputtering manner.

### SERS measurements

The SERS measurements were performed at room temperature on a confocal Raman spectrometer with the 514-nm laser focused to a diameter of 1 μm. The incident power was 0.55 mW, and the accumulation time was 10 s.

## Results

### Morphology of fabricated Au nanofilms

**Figure 1:**
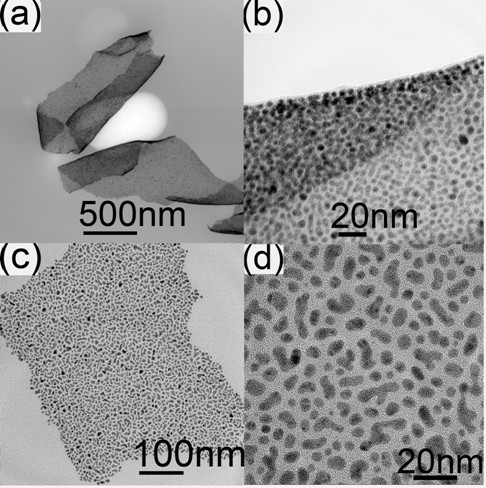
**TEM micrographs of the fabricated gold continuous nanofilms.** The four panels (**a**, **b**, **c**, **d**) highlight from different perspectives that the fabricated gold nanofilms are ultrathin continuous films.

Figure 1 shows the morphology of fabricated continuous ultrathin gold nanofilms. From Figure 1a,b, the folded nanofilms can be clearly seen as continuous and flexible, and their thickness is about 2 nm. From Figure 1c,d, we know that the nanofilms are composed of gold nanoparticle random arrays with uniform size, steady link, and ultrathin structure. Within the film, the size of the gold nanoparticles is only about 10 nm. The distance between nanoparticles is in sub-10 nm, filled with even thinner amorphous gold, which can be observed from the high-resolution transmission electron microscopy (TEM) images shown in Figure 1b,d.

### UV–vis absorption spectrum of the Au nanofilm layer on the ITO glass substrates

**Figure 2:**
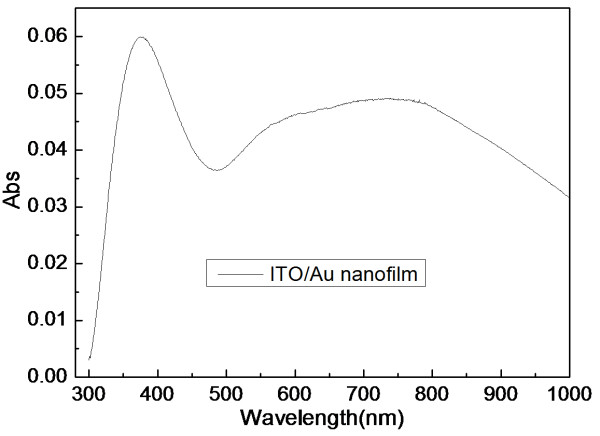
Visible absorption spectrum of the continuous Au nanofilm on the ITO glass substrate.

The localized absorption characteristic of Au films is highly sensitive to the surrounding medium, particle size, surface structure, and shape. The ultrathin Au nanofilm on the ITO glass substrate exhibits an ultraviolet–visible (UV–vis) optical spectrum in Figure 2. The continuous and inhomogeneous nanofilm, with a thickness of 2 nm or so and composed of nanometer-sized metal clusters, exhibits absorption in the UV–vis region attributed to the surface plasmon resonance in the metal islands. It is well known that optical absorption of island films of gold is a function of island density [26]. The absorption band resulting from bounded plasma resonance in the nanoparticles is shifted to longer wavelengths as the nanoisland density increases. The plasmonic absorption band is broadened due to a wider particle size distribution.

### The effect of UV–vis absorption spectra of the organic photosensitive layer incorporated in thin Au film

**Figure 3:**
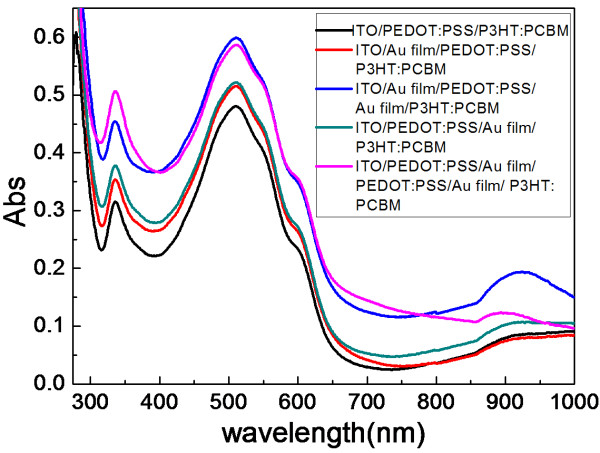
UV–vis absorption spectra of the blend films of P3HT:PCBM on ITO glass substrates.

Plasmonic enhancement of the P3HT:PCBM bulk heterojunction system is demonstrated in a spin-cast device with an incorporated ultrathin gold nanofilm thickness of 2 nm or so. Figure 3 exhibits the absorbance of P3HT:PCBM blend films with and without a layer of nanofilms. An enhanced optical absorption is observed in the spectral range of 350 to 1,000 nm where the P3HT:PCBM blend film is absorbing. The above results indicate that the enhanced absorption is due to the increased electric field in the plasmon photoactive layer by excited localized surface plasmons around the metallic nanoparticles. This enhancement is attributed to photon scattering and trapping by the surface plasmon generated in the metallic nanoparticles.

### The SERS spectra of R6G adsorbed on the surface of the Au nanofilm/glass

**Figure 4:**
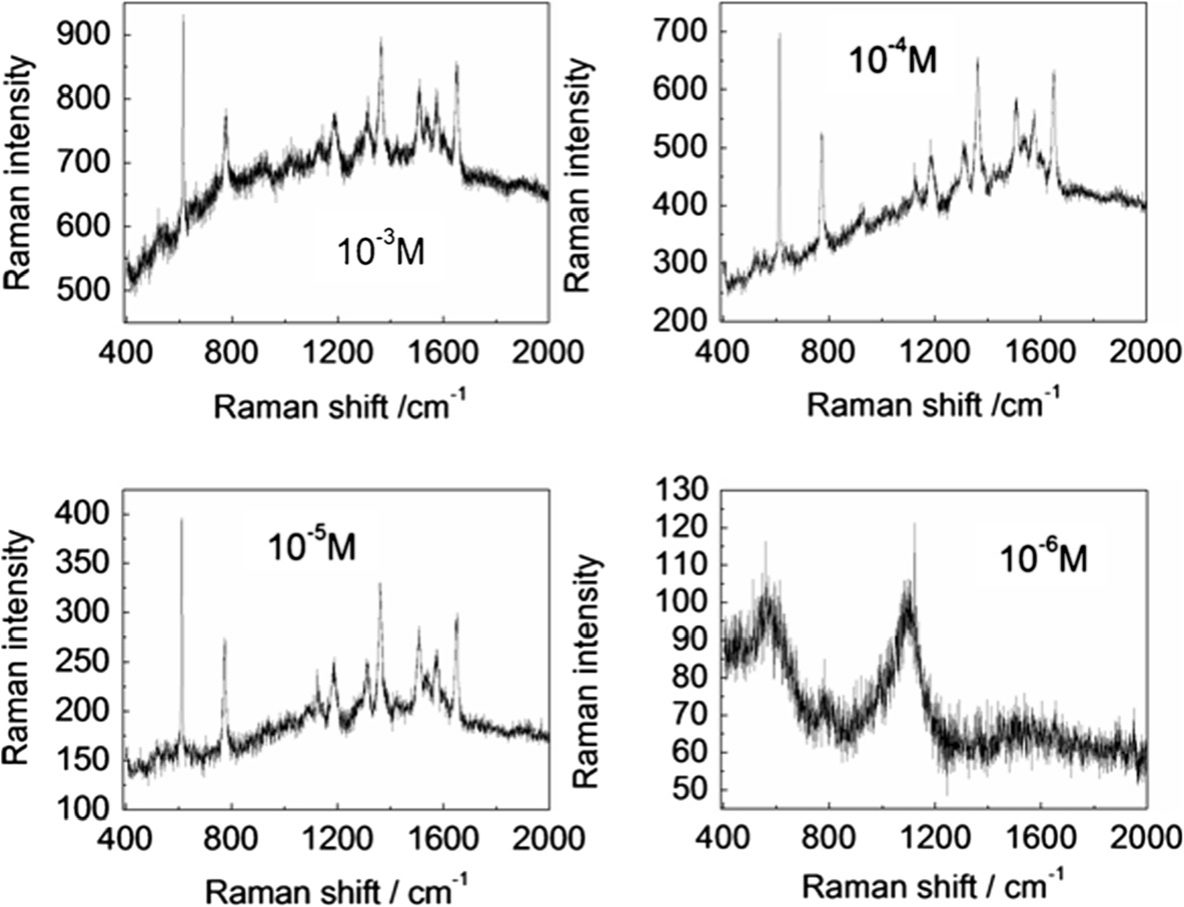
SERS spectra of R6G adsorbed on the surface of the Au nanofilm/glass.

Figure 4 shows a collection of spectra illustrating the Raman-enhancing capability. Many salient Raman peaks can be observed from the Rhodamine 6G (R6G) probe [27]. In comparison, different molar concentrations of R6G adsorbed on nanogold films shows a collection of spectra illustrating the efficiency of the SERS. As the molar concentration of R6G decreases, the intensity of the Raman spectra decreases. The junctions between the aggregated nanoparticles or nanoislands are believed to be SERS ‘hot spots’ where large field enhancements down to a single molecule are observed [28,29]. This is the result of localized surface plasmon resonance coupled between the nanoparticles and enhanced electromagnetic field intensity localized at the nanoparticle junctions [30].

## Discussion

To compare the impact of continuous ultrathin gold nanofilms on the absorption of visible light, plasmonic enhancement of the P3HT:PCBM bulk heterojunction system is demonstrated in a spin-cast device with an incorporated continuous ultrathin gold nanofilm thicknesses of 2 nm or so which are chosen to be sufficiently thin to limit the amount of light absorbed before reaching the active layer. The nanofilm incorporated with gold in the active P3HT:PCBM layer is shown to have significantly greater absorbance enhancement than the nanofilm without gold in the entire excitation spectral range in Figure 3. As shown in Figure 2, the optical absorption spectrum of the continuous ultrathin gold nanofilm has high light transmittance and broad surface plasmon resonance band in the wavelength range of 300 to 1,000 nm. Therefore, the results demonstrate that the enhancement of absorption in the wavelength range of 350 to 1,000 nm is due to the surface plasmon resonance absorption. The much higher plasma frequency of Au ensures a better overlap between plasmon resonance and absorption band of organic semiconductors. The light energy is trapped mainly in the P3HT:PCBM layer, leading to enhanced absorption in the active layer.

For the ITO/Au film/PEDOT:PSS/Au film/P3HT:PCBM and ITO/PEDOT:PSS/Au film/PEDOT:PSS/Au film/P3HT:PCBM structures, the plasmon resonance is located at a wavelength range of 350 to 1,000 nm. The plasmonic peak better overlaps the P3HT:PCBM absorption band. These enhancements concerning light absorption in the visible region can be explained by the surface plasmon polariton resonance of metallic nanoparticles in the gold nanofilm. When metallic nanoparticles are in close proximity, their plasmon resonances couple with each other and generate a light-scattering spectrum that depends strongly on the interparticle distance. The two-dimensional distinctive ultrathin continuous gold nanofilms can be used as subwavelength antennas in which the plasmonic near-field is coupled to the organic semiconductor, increasing its effective absorption cross section. A corrugated metallic film on the back surface of the P3HT:PCBM photosensitive layer can couple light into surface plasmon polariton (SPP) modes supported at the metal/P3HT:PCBM organic semiconductor interface as well as guided modes in the organic semiconductor slab. Both the shape and size of metal nanoparticles are key factors in determining the coupling efficiency. The two-layer ultrathin nanofilm increases the nanoparticle density; according to the Mie theory, the extinction coefficient is proportional to the nanoparticle density. Consequently, optical local-field enhancement of the two-layer continuous ultrathin gold nanofilm is stronger than that of the one-layer ultrathin continuous gold nanofilm. Figure 3 embodies the absorbance of the two-layer ultrathin continuous gold nanofilm which far outweighs that of ITO/PEDOT:PSS/Au film/P3HT:PCBM and ITO/Au film/PEDOT:PSS/P3HT:PCBM. In brief, the enhanced efficiency is shown to stem from field enhancement originating both from localized plasmonic resonances and periodic similar nanopatch antenna configuration and SPP modes in the peculiar gold nanofilm.

To investigate the performance for electromagnetic enhancement, SERS spectroscopic measurements were carried out using Rhodamine 6G, a well-characterized test molecule. Spectra obtained from Rhodamine 6G molecules at a concentration of 10^−3^ to 10^−6^ M are shown in Figure 4 which exhibit repeatable high SERS sensitivity. The distances between the centers of two adjacent particles and the particle diameter are important parameters affecting SERS activity. This ultrathin continuous gold nanofilm produces a high Raman signal due to its periodic arrangements, high nanoisland density, and control of the gap between the nanostructures in the sub-10-nm regime. The observed SERS efficiency can be explained in terms of interparticle coupling-induced Raman enhancement. Thus, the distinctive continuous gold nanofilm is very effective in providing abundant hot spots for SERS enhancement.

## Conclusions

In conclusion, we have produced continuous ultrathin gold nanofilms with high local-field enhancement effect and a high SERS activity. Spectral analysis suggests that the prominent light absorption in organic photosensitive materials and the high SERS activity arise from the near-field effect of localized surface plasmons of nanoparticles. Owing to their distinctive morphology and high light transmittance, continuous ultrathin gold nanofilms can be used in multilayer organic solar cells to trap light without affecting the physical thickness of solar photovoltaic absorber layers and yielding new options for solar cell design. Further work is needed to research two-dimensional distinctive continuous gold nanofilms that are utilized to trap light in solar cells which may be suitable for application to the high photoelectric conversion efficiency of organic solar cells.

## Competing interests

The authors declare that they have no competing interests.

## Authors’ contributions

L-DW carried out the design and the characterization of ultrathin gold films, performed the ultrathin gold nanofilm surface plasmon resonance analysis, and drafted the manuscript. R-ZL participated in the fabrication of gold films. S-QZ participated in the SERS measurement. TZ, X-YZ, Q-LW, and XL read the manuscript and contributed to its improvement. All authors read and approved the final manuscript.
